# Survey on hypoglycemia among insulin-treated patients with diabetes: The Colombian International Operations Hypoglycemia Assessment Tool population

**DOI:** 10.7705/biomedica.4365

**Published:** 2019-09-01

**Authors:** Ana M. Gómez, Luis G. Chica, Álvaro F. Burbano, Esdras M. Vásqueze, Jorge A. Escobar, Paola M. Arias, Dora I. Molina

**Affiliations:** 1 Departamento de Endocrinología, Hospital Universitario San Ignacio, Bogotá, D.C., Colombia Departamento de Endocrinología Hospital Universitario San Ignacio BogotáD.C Colombia; 2 Centro de Excelencia para el Manejo de la Diabetes (CEMDI), Bogotá, D.C., Colombia Centro de Excelencia para el Manejo de la Diabetes BogotáD.C Colombia; 3 Especialización en Medicina Familiar, Universidad El Bosque, Bogotá, D.C., Colombia Universidad El Bosque Especialización en Medicina Familiar Universidad El Bosque BogotáD.C Colombia; 4 Departamento de Endocrinología, Clínica Integral de Diabetes, Medellín, Colombia Departamento de Endocrinología Clínica Integral de Diabetes Medellín Colombia; 5 CMRQ, Novo Nordisk Colombia S.A.S., Bogotá, D.C., Colombia CMRQ Novo Nordisk Colombia S.A.S BogotáD.C Colombia; 6 Facultad de Ciencias de la Salud, Universidad de Caldas, Manizales, Colombia Universidad de Caldas Facultad de Ciencias de la Salud Universidad de Caldas Manizales Colombia; 7 IPS Médicos Internistas de Caldas, Manizales, Colombia IPS Médicos Internistas de Caldas Manizales Colombia

**Keywords:** Hypoglycemia, diabetes mellitus, insulin infusion systems, Colombia, hipoglucemia, diabetes mellitus, sistemas de infusión de insulina, Colombia

## Abstract

**Introduction:**

The non-interventional International Operations Hypoglycemia Assessment Tool (IO-HAT) study assessed the incidence of hypoglycemia in patients with insulin-treated diabetes across nine countries, including a cohort of patients in Colombia.

**Materials and methods:**

Hypoglycemia incidence among patients with insulin-treated diabetes was assessed across 26 sites in Colombia. Hypoglycaemic events (any, nocturnal or severe) were reported in self-assessment questionnaires (SAQ) and patient diaries based on capillary blood glucose measurement or symptoms. Retrospective events (severe events 6 months before baseline and any event 4 weeks before baseline) were recorded in SAQ, Part 1, and prospective events (4 weeks from baseline) were recorded in SAQ, Part 2, and patient diaries. Differences in hypoglycemia incidence reported in the retrospective and prospective periods were assessed using two-sided tests.

**Results:**

Of the 664 patients assessed, 213 had type 1 diabetes (T1D) and 451 had type 2 diabetes (T2D). Nearly all patients experienced at least one hypoglycaemic event in the prospective period (97.1% T1D; 93.3% T2D). Rates of hypoglycemia (events per person- year, PPY) were higher prospectively than retrospectively for any hypoglycemia (T1 D: 121.6 vs. 83.2, p<0.001; T2D: 28.1 vs. 24.6, p=0.127) and severe hypoglycemia (T 1D: 15.3 vs. 9.2, p=0.605; T 2 D: 9.5 vs. 3.5 p=0.040).

**Conclusion:**

These results, the first from a patient-reported dataset on hypoglycemia in insulin-treated patients with diabetes in Colombia, show that patients reported higher rates of any hypoglycemia during the prospective period.

## Resumen

**Introducción.:**

En el estudio no intervencionista *International Operations Hypoglycemia Assessment Tool* (IO-HAT), se evalúo la incidencia de hipoglucemia en pacientes diabéticos tratados con insulina en nueve países, incluido Colombia.

**Materiales y métodos.:**

La incidencia de hipoglucemia entre pacientes diabéticos tratados con insulina se evaluó en 26 centros médicos en Colombia. Los episodios de hipoglucemia determinados con base en la medición de la glucemia capilar o en los síntomas se reportaron en el cuestionario de autoevaluación (*Self-Assessment Questionnaire,* SAQ) y en el diario del paciente. Los episodios retrospectivos (episodios graves y cualquiera ocurrido 6 meses y 4 semanas antes del inicio del estudio, respectivamente) se registraron en el SAQ, parte 1, y los eventos prospectivos (4 semanas desde el inicio), en el SAQ, parte 2, y en el diario del paciente. Las diferencias en la incidencia de la hipoglucemia entre los períodos retrospectivo y prospectivo se evaluaron mediante una prueba de dos colas.

**Resultados.:**

De los 664 pacientes evaluados, 213 tenían diabetes de tipo 1 y 451 tenían diabetes de tipo 2. Casi todos los pacientes experimentaron al menos un episodio de hipoglucemia en el período prospectivo (97,1 %, diabetes de tipo 1, y 93,3 %, diabetes de tipo 2). Los índices de hipoglucemia (episodios año-persona) fueron mayores prospectivamente que retrospectivamente para cualquier tipo de hipoglucemia (diabetes de tipo 1: 121,6 *Vs.* 83,2; p<0,001; la diabetes de tipo 2: 28,1 *Vs.* 24,6; p=0,127) y para la hipoglucemia grave (diabetes de tipo 1: 15,3 *Vs.* 9,2; p=0,605; diabetes de tipo 2: 9,5 *Vs.* 3,5; p=0,040).

**Conclusión.:**

Estos resultados, que constituyen el primer conjunto de datos sobre hipoglucemia informados por pacientes diabéticos colombianos tratados con insulina, evidenciaron tasas más altas para ambos tipos de hipoglucemia durante el período prospectivo.

In 2010, the overall prevalence of diabetes in Colombia was reported to be 4-8% depending on the age range of the population studied ^1^. As with many countries around the world, this prevalence is increasing and more recently, the International Diabetes Federation (IDF) estimated there were over 3 million adults with diabetes in Colombia in 2015 with a national prevalence of 9.6% among adults aged 20-79 years and an annual average diabetes-related cost per person of USD$ 772.9 ^2^. It is estimated that the number of people with diabetes in Latin America will increase by 148% between 2000 and 2030 ^1^.

The key goal in the management of diabetes is to maintain normal blood glucose levels. Insulin therapy is used in the treatment of type 1 diabetes (T1D), and less commonly in the treatment of type 2 diabetes (T2D). Hypoglycemia is a frequent side effect of insulin therapy and is a major limiting factor in achieving good glycaemic control ^3^. This condition impacts the patient’s quality of life ^4^ and can result in increased morbidity ^3^ and mortality ^5^. Besides, its treatment represents a significant burden on the healthcare system ^6^.

Data on hypoglycemia rates in real-world practice, particularly in developing countries, are limited. Randomized clinical trials (RCT) often exclude patients with comorbidities or frequent episodes of hypoglycemia, which means that hypoglycemia rates reported from these RCTs are not representative of those in everyday clinical practice ^7^.

The global Hypoglycemia Assessment Tool (HAT) study, which was designed to determine the incidence of hypoglycemia in a global insulin-treated patient population, demonstrated that the real-world incidence of hypoglycemia is high compared with rates reported from randomized clinical trials. The study also showed there are regional differences in hypoglycemia incidence, with the highest rates being observed among patients with T1D in Latin America ^8^.

The Global Attitude of Patients and Physicians 2 (GAPP2) survey, which evaluated data on self-treated hypoglycemia in patients with T2D in seven countries ^9^, reported that self-treated hypoglycemia in Argentina was relatively common in patients using basal insulin analogs -at least one event in the previous 30 days was reported by 31% of patients with diabetes ^9^. In the Brazilian cohort of the HAT study, 91.7% of patients with T1D and 61.8% of patients with T2D reported at least one hypoglycaemic event during the 4-week prospective observation period ^10^.

The International Operations (IO) HAT (IO-HAT) study builds on the information collected from the global HAT study and was designed to assess the incidence of hypoglycemia in patients with T1D or T2D treated with insulin (premix, short-acting, long-acting or sensor-augmented pump [SAP]) in Bangladesh, Colombia, Egypt, Indonesia, Philippines, Singapore, South Africa, Turkey, and the United Arab Emirates. The current analysis evaluates data from the Colombian cohort of insulin-treated patients with diabetes enrolled in the IO-HAT study.

## Materials and methods

This was an international, multicentre non-interventional, 6-month retrospective and 4-week prospective study of hypoglycaemic events conducted across 26 sites in Colombia using self-assessment questionnaires (SAQ) and patient diaries. The study was conducted in accordance with the Declaration of Helsinki ^11^ and the Guidelines for Good Pharmacoepidemiology Practices ^12^ and it was approved by country- specific regulatory and ethics agencies as applicable.

Study population

Eligible patients were enrolled consecutively during a routinely scheduled clinical consultation with their healthcare provider. Patients were eligible for the study if they were ≥18 years of age at baseline, had T1D/T2D treated with insulin for >12 months and had provided informed consent. Patients were excluded from the study if they were non-ambulatory, illiterate or otherwise unable to complete the written survey.

Study endpoints

The primary endpoint of the study was to determine the percentage of patients who experience at least one hypoglycaemic episode during the 4-week prospective observational period among the insulin-treated patients with T1D or T2D.

Secondary endpoints included the incidence of hypoglycemic events, the relationship between patient demography, diabetes treatment, and the incidence of hypoglycemic episodes. The use of health system resources (number of hospital admission days, additional clinic appointments, and telephone contacts as a result of hypoglycaemic episodes) was assessed. The impact on patient behaviors as a result of hypoglycemia was assessed by the number of consultations with a doctor or nurse, increased calorie intake, avoidance of physical exercise, reduced or skipped insulin doses, increase in the frequency of blood glucose monitoring as a result of fear of hypoglycemia (continuous on scale of 0-10 where 0 is not afraid at all and 10 is absolutely terrified) or hypoglycaemic episodes, and any sick leave, sick days or short days as a result of hypoglycaemic episodes.

Assessments

The assessment was by a two-part SAQ consisting of a retrospective cross- sectional evaluation (Part 1) and a prospective observational evaluation (Part 2). Patient diaries were also provided to assist recall and to record hypoglycemic events, the effect of hypoglycemia on productivity, and healthcare utilization and productivity over the 4 weeks following study entry. The SAQs used for IO-HAT were similar to those used in the global HAT study with modifications to collect additional data on variables such as comorbidities, type of diabetes treatment used, loss of productivity, and quality of life. Paired responses to the Part 1 and Part 2 SAQs were used to estimate any differences in reporting of hypoglycemia between the retrospective and prospective periods.

Hypoglycemia classification

Severe hypoglycemia was defined as a hypoglycaemic event requiring third party assistance, which is consistent with the American Diabetes Association (ADA) definition ^13^.

Non-severe hypoglycemia was defined as an episode managed by the patient alone.

Any hypoglycemic event was defined as the sum of severe and non- severe hypoglycemia.

Nocturnal hypoglycemia was defined as hypoglycemia occurring between midnight and 06:00 h.

A combined measure of any hypoglycemia (based on the sum of all hypoglycaemic events) was derived from both the patient diary and SAQ entries.

Hypoglycemia awareness

Hypoglycemia awareness (as defined by Pedersen ^14^ and as per protocol definition) was evaluated using the responses to the question: “How often do you have symptoms when you have a low blood sugar measurement?” and where the response “always” or “usually” denoted ‘normal’ hypoglycemia awareness, “occasionally” denoted ‘impaired’ awareness, and “never” denoted ‘severely impaired’ awareness.

Statistical analysis

All statistical tests were two-sided and regarded as exploratory. Statistical significance was set at p<0.05 and no adjustments were made for multiple comparisons. The percentage of patients experiencing at least one hypoglycaemic event during the observation period was calculated together with the 95% confidence interval (CI) for this percentage. Binomial distribution was assumed. Data are presented as mean (SD) unless otherwise stated.

Ethical considerations

The study protocol and assessments were conducted according to the Declaration of Helsinki and the International Conference on Harmonisation Good Clinical Practice Guidelines. The protocol was approved by independent ethics committees or institutional review boards before the start of the study. Signed informed consent was obtained from each patient before any study- related activities. All study materials were translated into Spanish and the data obtained were translated back into English for analysis.

## Results

Patient characteristics

In the Colombian cohort, 664 patients completed the SAQ, Part 1, and were included in the full analysis set; 657 patients completed SAQ, Part 2, and were included in the completers analysis set; 653 patients completed patient diaries. Of the 664 patients completing the SAQ, Part 1, 213 had T1D and 451 had T2D. Patient characteristics are shown in table 1. Overall, 35.3% of patients were male. Patients with T2D were older than those with T1D (mean age, 63.2 years vs. 36.0 years, respectively). The duration of diabetes was longer in patients with T1D than T2D (17.2 years vs. 14.4 years). At baseline, patients with T1D had better levels of glycemic control than patients with T2D (HbA1c 7.7% in T1D and 8.4% in T2D).

**Table 1:** Characteristics of the patient population

Number of patients who completed part 2 of SAQ, n (%)	211 (99.1)	446 (98.9)
Sex: male/female, %	32.9/65.7	37.7/61.4
Age, years	36.0 (13.6)	63.2 (10.9)
BMI (kg/m2)	24.7 (3.8)	28.4 (4.9)
Duration of diabetes, years	17.2 (10.1)	14.4 (8.7)
Duration of insulin use, years	16.1 (10.1)	6.8 (5.9)
HbA1c, %*	7.7 (1.4)	8.4 (1.8)
FBG, mg/dl	154.8 (64.8)	136.8 (50.4)
PPG, mg/dl	153.0 (77.4)	180.0 (72.0)
Previous medical illnesses, n (%*)		
Neuropathy	43 (20.2)	194 (43.0)
Retinopathy	47 (22.1)	181 (40.1)
Peripheral vascular disease	11 (5.2)	97 (21.5)
Nephropathy	41 (19.2)	131 (29.0)
Myocardial infarction	5 (2.3)	58 (12.9)
Angina	5 (2.3)	44 (9.8)
None}	122 (57.3)	124 (27.5)
Method of diabetes treatment, n (%)		
Short-acting insulin	97 (45.5)	15 (3.3)
Long-acting insulin	8 (3.8)	173 (38.4)
Pre-mix	0	4 (0.9)
Both short and long acting	105 (49.3)	250 (55.4)
Both short acting and pre-mix	0	5 (1.1)
Both long acting and pre-mix	1 (0.5)	3 (0.7)
Short and long acting and pre-mix	0	0
Missing	2 (0.9)	1 (0.2)
Use of insulin pump, n (%)		
Yes	97 (45.5)	17 (3.8)
No	112 (52.6)	434 (96.2)
Other	4 (1.9)	0 (0)
Checks blood sugar levels, n (%)		
Yes	207 (97.2)	434 (96.2)
No	4 (1.9)	12 (2.7)
Not sure	1 (0.5)	4 (0.9)

BMI: body mass index; FBG: fasting blood glucose; n: total number of subjects participating; PPG: postprandial glucose; SAQ: self-assessment questionnaire; SD: standard deviation; T1D: type 1 diabetes; T2D: type 2 diabetes

Data expressed as mean (SD) unless otherwise stated.

* Percentages based on number of patients with evaluable data

Hypoglycemia

**Figure 1:**
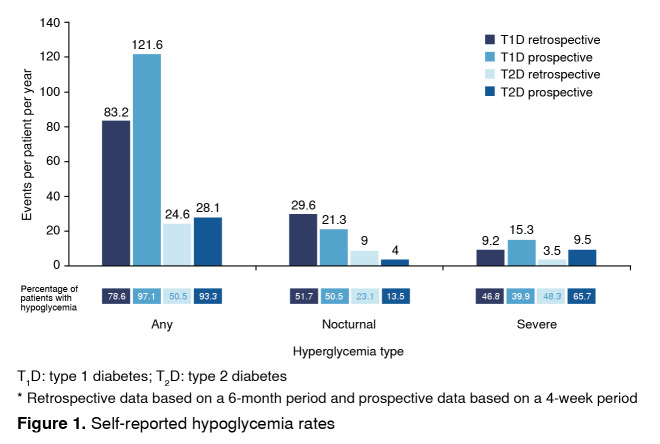
Self-reported hypoglycemia rates

Nearly all patients experienced at least one hypoglycaemic event in the 4-week prospective observation period (97.1% of patients with T1D and 93.3% of those with T2D). The proportion of patients reporting a hypoglycaemic event in the prospective and retrospective periods is shown in figure 1. Hypoglycaemic rates (estimated number of events per patient-year [PPY]) of any and severe hypoglycemia were higher in the prospective period than in the retrospective period (figure 1).

The estimated incidence rate ratio (RR) for any hypoglycemia in the prospective and retrospective periods was 1.46 (p<0.001) for T1D and 1.14 (p=0.127) for T2D. The proportion of patients reporting nocturnal hypoglycemia was higher retrospectively than prospectively in patients with T1D (51.7% vs. 50.5%) and T2D (23.1% vs. 13.5%). Nocturnal hypoglycemia rates were higher retrospectively in patients with T1D (29.6 events PPY vs. 21.3 events PPY, RR=0.72; p=0.036) and T2D (9.0 events PPY vs. 4.0 events PPY, RR=0.43; p<0.001). The proportion of patients who defined hypoglycemia by using both blood glucose measurement and symptoms was 49.3% in T1D and 30.6% in T2D.

Hypoglycemia incidence by insulin regimen

**Figure 2:**
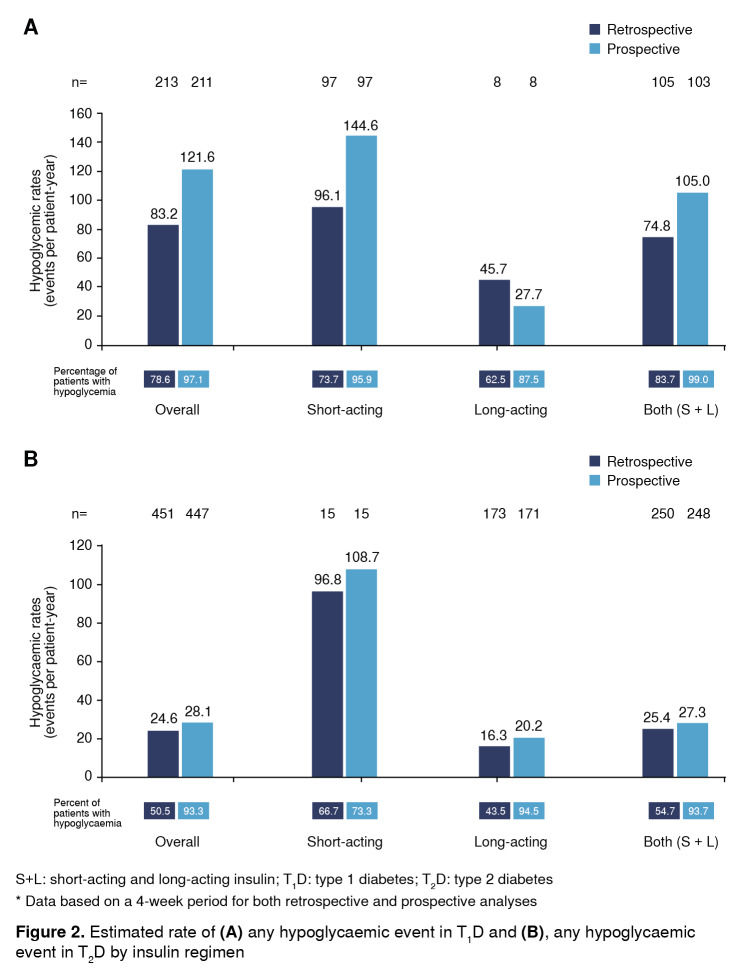
Estimated rate of **(A)** any hypoglycaemic event in T1D and **(B)**, any hypoglycaemic event in T2D by insulin regimen

The incidence rates for any hypoglycemia by insulin regimen are shown in figure 2 A and B.

Healthcare utilization

**Figure 3:**
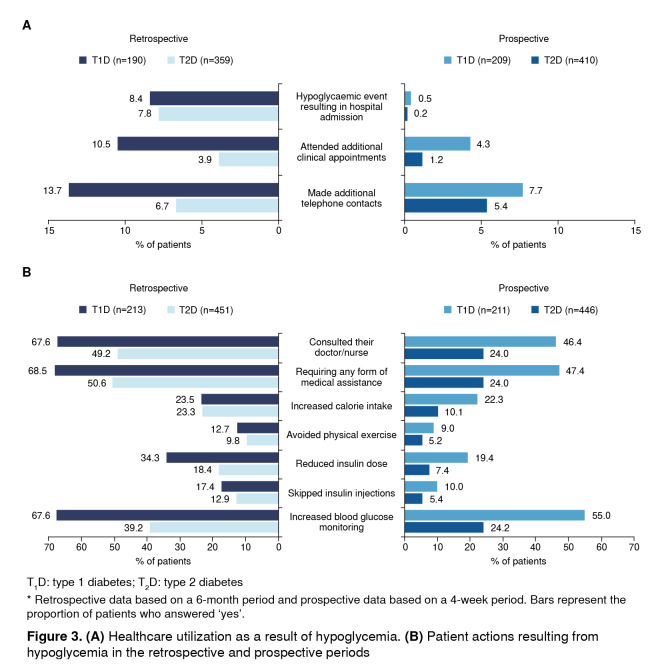
(A) Healthcare utilization as a result of hypoglycemia. (B) Patient actions resulting from hypoglycemia in the retrospective and prospective periods

Healthcare utilization as a result of hypoglycemia in the 6-month retrospective and 4-week prospective period is shown in figure 3 A. Healthcare utilization was higher in the retrospective period than in the prospective period both in patients with T1D and T2D. In the prospective period, healthcare utilization (admission to hospital, additional clinic appointments, and telephone contact as a result of hypoglycemic episodes) was higher in patients with T1D than T2D.

Patient knowledge and behaviors

More patients with T1D than T2D indicated they had knowledge of hypoglycemia before reading the definition in the Part 1, SAQ (97.5% vs. 78.7%, respectively). The proportion of patients who measured blood sugar to determine hypoglycemia but provided values that were inconsistent with standard definitions (≤3.9 mmol/L or ≤70 mg/dL) was higher in patients with T2D (12.9%) than those with T1D (4.9%). Patients with T1D had more hypoglycemia awareness than patients with T2D. Normal awareness was reported in 60.1% (T1D) vs. 32.4% (T2D) of patients, impaired awareness was reported by 37.6% (T1D) vs. 50.8% (T2D) and severely impaired awareness by 2.3% (T1D) vs. 12.0% (T2D).

Patient actions as a result of hypoglycemia are shown in figure 3B. Patients with T1D were more likely than those with T2D to increase their blood glucose monitoring following a hypoglycaemic episode. With respect to fear of hypoglycemia, there were no notable differences between patients with T1D [mean (SD) score, 6.9 (2.98)] and T2D [mean (SD) score, 6.2 (3.28)].

## Discussion

The prevalence of diabetes in Colombia is approximately 9.6% and this is estimated to rise to 12.9% by 2040 ^2^. Diabetes is the fifth leading cause of mortality in the country and the disease is also associated with high morbidity ^15^. Incidence of hypoglycemia is high in the Latin America region; the GAPP2 survey estimated that 31% of patients with diabetes in Argentina experienced at least one hypoglycaemic event in the previous 30 days ^9^, whilst the Brazilian cohort of the HAT study reported 91.7% of patients with T1D and 61.8% of patients with T2D had experienced at least one hypoglycaemic event during the 4-week prospective observation period of the study ^10^.

In the current study, patients reported higher rates of overall and severe hypoglycemia during the prospective period. The difference in rates between the two periods may be a result of recall bias as patient diaries were provided to assist recall and record hypoglycaemic events during the prospective period whilst the retrospective data were based on the SAQ. In contrast, patients reported higher nocturnal rates of hypoglycemia in the retrospective period. It is possible that the definition of nocturnal hypoglycemia (between midnight and 06:00 h) may have been adhered to more strictly during the prospective period than the retrospective period. Patients may also have reported more episodes of nocturnal hypoglycemia retrospectively owing to recall bias and the belief that these events occur more frequently than they do. Furthermore, nocturnal hypoglycemia may be underreported as the patient diaries may be more difficult to complete at night than during the day. Overall, the results of our study suggest that the incidence of hypoglycemia may be underreported.

The incidence of hypoglycemia (all, nocturnal, and severe) in Colombian patients was high compared with the IO-HAT overall results (Emral, *et al.,* in progress). It should be noted that Colombia is the only country which specifically categorized patients with T1D or T2D using insulin pumps. The high incidence of hypoglycemia in this cohort may be primarily driven by the use of insulin pumps with integrated continuous glucose monitoring, as patients are able to see their glucose readings constantly and, therefore, notice and report more hypoglycemia. Besides, as readings from pumps are not 100% accurate, the possibility of falsely low value (even though the patient is euglycemic) cannot be ruled out ^16^.

Colombia has one of the highest prevalence of adults with diabetes in South and Central America ^17^. Poor glycemic control, defined as HbA1c >7%, is a strong predictor of complications that result in increased resource usage within the region. One study assessing the quality of diabetic care in Colombia reported that only 42.9% of patients had HbA1c levels ≤7%, whilst 21.1% of patients had HbA1c >9% ^18^. Mean HbA1c levels in the current study were >7% at baseline indicating poor glycaemic control. No association between HbA1c and hypoglycemia was identified in the current study, which suggests that hypoglycemia is common at all levels of glycaemic control.

Hypoglycemia influences treatment compliance, which can result in an increase in complications due to lack of disease control as a result of modifying the insulin dose in response to hypoglycemia. In a 7-year study evaluating consistency in using diabetic medications in a Mexican American population, inconsistent use of medication was associated with an increased risk in kidney problems and death ^19^.

Patient actions as a result of hypoglycemia lead to an increase in the use of healthcare resources and the health economic burden ^6^. In particular, severe hypoglycaemic events often result in emergency/ambulance calls and hospital treatment, thereby incurring in substantial healthcare costs ^20^. The proportion of patients seeking medical advice or requiring hospitalization as a result of hypoglycemia was lower during the prospective period of the IO-HAT Colombian study, which may be explained by increased patient knowledge as a result of having participated in the study.

Our results suggest that increasing patient knowledge of hypoglycemia may reduce its impact on the healthcare system and enable patients to meet their individual treatment goals more effectively. Furthermore, the results of this study highlight the need for patient education regarding hypoglycemia and for actively promoting hypoglycaemic control in clinical practice.

As with any observational study, there are strengths and limitations to IO-HAT. Limitations include the short follow-up period of the study and the possibility of bias since hypoglycemia is self-reported by the patient based on patient recall of information, which may be less accurate for the retrospective period. However, this is also a strength of the study, as self-reporting captures information such as missed blood glucose tests or unawareness of the threshold at which blood glucose concentrations represent a hypoglycaemic event. In addition, the observational nature of the study means that the results reflect real-life practice.

Insulin is the mainstay of diabetes treatment, however, hypoglycemia remains a major obstacle in achieving good glycaemic control and fear of hypoglycemia can limit treatment intensification as a result of the patient’s unwillingness to take medication. New technologies, in particular the new generation longer-acting basal insulins have been shown to have significantly more predictable glucose-lowering effects and may help to lower the risk of hypoglycemia without compromising efficacy ^21^.

In conclusion, these results are the first patient-reported dataset on hypoglycemia in Colombia. Patients reported higher rates of hypoglycemia (any and severe) during the prospective period. This could be a result of recall bias during the retrospective study period or of increased patient knowledge on hypoglycemia due to their participation in the study. The results of our study are in line with the overall data from the IO-HAT study and indicate that hypoglycemia is underreported and, therefore, underestimated.

## References

[1] Aschner P (2010). Epidemiología de la diabetes en Colombia. Avances en Diabetología.

[2] Federation ID (2016). IDF Diabetes Atlas.

[3] Barnett AH, Cradock S, Fisher M, Hall G, Hughes E, Middleton A (2010). Key considerations around the risks and consequences of hypoglycemia in people with type 2 diabetes. Int J Clin Pract.

[4] Davis RE, Morrissey M, Peters JR, Wittrup-Jensen K, Kennedy-Martin T, Currie CJ (2005). Impact of hypoglycemia on quality of life and productivity in type 1 and type 2 diabetes. Curr Med Res Opin.

[5] Bonds DE, Miller ME, Bergenstal RM, Buse JB, Byington RP, Cutler JA (2010). The association between symptomatic, severe hypoglycemia and mortality in type 2 diabetes: Retrospective epidemiological analysis of the ACCORD study. BMJ.

[6] Brod M, Wolden M, Christensen T, Bushnell DM (2013). Understanding the economic burden of nonsevere nocturnal hypoglycemic events: Impact on work productivity, disease management, and resource utilization. Value Health.

[7] Elliott L, Fidler C, Ditchfield A, Stissing T (2016). Hypoglycemia event rates: A comparison between real-world data and randomized controlled trial populations in insulin-treated diabetes. Diabetes Ther.

[8] Khunti K, Alsifri S, Aronson R, Cigrovski Berkovic M, Enters-Weijnen C, Forsen T (2016). Rates and predictors of hypoglycemia in 27 585 people from 24 countries with insulin- treated type 1 and type 2 diabetes: The global HAT study. Diabetes Obes Metab.

[9] Brod M, Galstyan G, Unnikrishnan AG, Harman-Boehm I, Prusty V, Lavalle F (2016). Self- treated hypoglycemia in type 2 diabetes mellitus: Results from the second wave of an international cross-sectional survey. Diabetes Ther.

[10] Lamounier RN, Geloneze B, Oliveira Leite S, Jr. R Montenegro, Zajdenverg L, Fernandes M (2018). Hypoglycemia incidence and awareness among insulin-treated patients with diabetes: The HAT study in Brazil. Diabetol Metab Syndr.

[11] World Medical Association (2013). Declaration of Helsinki: Ethical principles for medical research involving human subjects. JAMA.

[12] International Society for Pharmacoepidemiology (2008). Guidelines for good pharmacoepidemiology practices (GPP). Pharmacoepidemiol Drug Saf.

[13] American Diabetes Association (2005). Defining and reporting hypoglycemia in diabetes: A report from the American Diabetes Association Workgroup on Hypoglycemia. Diabetes Care.

[14] Pedersen-Bjergaard U, Pramming S, Thorsteinsson B (2003). Recall of severe hypoglycaemia and self-estimated state of awareness in type 1 diabetes. Diabetes Metab Res Rev.

[15] Vargas-Uricoechea H, Casas-Figueroa LA (2015). An epidemiologic analysis of diabetes in Colombia. Ann Glob Health.

[16] Gómez AM, Marín-Sánchez A, Muñoz OM, Colón-Peña CA (2015). Numerical and clinical precision of continuous glucose monitoring in Colombian patients treated with insulin infusion pump with automated suspension in hypoglycemia. Endocrinol Nutr.

[17] Aschner P, Aguilar-Salinas C, Aguirre L, Franco L, Gagliardino JJ, de Lapertosa SG (2014). Diabetes in South and Central America: An update. Diabetes Res Clin Prac.

[18] Machado-Alba JE, Moncada-Escobar JC, Gaviria H (2009). Quality and effectiveness of diabetes care for a group of patients in Colombia. Rev Panam Salud Pública.

[19] Kuo YF, Raji MA, Markides KS, Ray LA, Espino DV, Goodwin JS (2003). Inconsistent use of diabetes medications, diabetes complications, and mortality in older mexican americans over a 7-year period: Data from the Hispanic established population for the epidemiologic study of the elderly. Diabetes Care.

[20] Heller SR, Frier BM, Hersløv ML, Gundgaard J, Gough SC (2016). Severe hypoglycaemia in adults with insulin-treated diabetes: Impact on healthcare resources. Diabetic Med.

[21] Heise T, Hermanski L, Nosek L, Feldman A, Rasmussen S, Haahr H (2012). Insulin degludec: Four times lower pharmacodynamic variability than insulin glargine under steady-state conditions in type 1 diabetes. Diabetes Obes Metab.

